# Causal Artificial Intelligence Models of Food Quality Data

**DOI:** 10.17113/ftb.62.01.24.8301

**Published:** 2024-03

**Authors:** Želimir Kurtanjek

**Affiliations:** University of Zagreb Faculty of Food Technology and Biotechnology, Pierotijeva 6, 10000 Zagreb, Croatia

**Keywords:** Bayesian network, AI causality, intervention effects, ACE, food quality

## Abstract

**Research background:**

The aim of this study is to emphasize the importance of artificial intelligence (AI) and causality modelling of food quality and analysis with ’big data’. AI with structural causal modelling (SCM), based on Bayesian networks and deep learning, enables the integration of theoretical field knowledge in food technology with process production, physicochemical analytics and consumer organoleptic assessments. Food products have complex nature and data are highly dimensional, with intricate interrelations (correlations) that are difficult to relate to consumer sensory perception of food quality. Standard regression modelling techniques such as multiple ordinary least squares (OLS) and partial least squares (PLS) are effectively applied for the prediction by linear interpolations of observed data under cross-sectional stationary conditions. Upgrading linear regression models by machine learning (ML) accounts for nonlinear relations and reveals functional patterns, but is prone to confounding and failed predictions under unobserved nonstationary conditions. Confounding of data variables is the main obstacle to applications of the regression models in food innovations under previously untrained conditions. Hence, this manuscript focuses on applying causal graphical models with Bayesian networks to infer causal relationships and intervention effects between process variables and consumer sensory assessment of food quality.

**Experimental approach:**

This study is based on the data available in the literature on the process of wheat bread baking quality, consumer sensory quality assessments of fermented milk products, and professional wine tasting data. The data for wheat baking quality were regularized by the least absolute shrinkage and selection operator (LASSO elastic net). Bayesian statistics was applied for the evaluation of the model joint probability function for inferring the network structure and parameters. The obtained SCMs are presented as directed acyclic graphs (DAG). D-separation criteria were applied to block confounding effects in estimating direct and total causal effects of process variables and consumer perception on food quality. Probability distributions of causal effects of the intervention of individual process variables on quality are presented as partial dependency plots determined by Bayesian neural networks. In the case of wine quality causality, the total causal effects determined by SCMs are positively validated by the double machine learning (DML) algorithm.

**Results and conclusions:**

The data set of 45 continuous variables corresponding to different chemical, physical and biochemical variables of wheat properties from seven Croatian cultivars during two years of controlled cultivation were analysed. LASSO regularization of the data set yielded the ten key predictors, accounting for 98 % variance of the baking quality data. Based on the key variables, the quality predictive random forest model with 75 % cross-validation accuracy was derived. Causal analysis between the quality and key predictors was based on the Bayesian model shown as a DAG graph. Protein content shows the most important direct causal effect with the corresponding path coefficient of 0.71, and THMM (total high-molecular-mass glutenin subunits) content was an indirect cause with a path coefficient of 0.42, and protein total average causal effect (ACE) was 0.65. The large data set of the quality of fermented milk products included binary consumer sensory data (taste, odour, turbidity), continuous physical variables (temperature, fat, pH, colour) and three grade classes of products by consumer quality assessment. A random forest model was derived for the prediction of the quality classification with an out-of-bag (OOB) error of 0.28 %. The Bayesian network model predicts that the direct causes of the taste classification are temperature, colour and fat content, while the direct causes of the quality classification are temperature, turbidity, odour and fat content. The key quality grade ACE of temperature -0.04 grade/°C and 0.3 quality grade/fat content were estimated. The temperature ACE dependency shows a nonlinear type as negative saturation with the ’breaking’ point at 60 °C, while for fat ACE had a positive linear trend. Causal quality analysis of red and white wine was based on the large data set of eleven continuous variables of physical and chemical properties and quality assessments classified in ten classes, from 1 to 10. Each classification was obtained in triplicate by a panel of professional wine tasters. A non-structural double machine learning (DML) algorithm was applied for total ACE quality assessment. The alcohol content of red and white wine had the key positive ACE relative factor of 0.35 quality/alcohol, while volatile acidity had the key negative ACE of –0.2 quality/acidity. The obtained ACE predictions by the unstructured DML algorithm are in close agreement with the ACE obtained by the structural SCM.

**Novelty and scientific contribution:**

Novel methodologies and results for the application of causal artificial intelligence models in the analysis of consumer assessment of the quality of food products are presented. The application of Bayesian network structural causal models (SCM) enables the d-separation of pronounced effects of confounding between parameters in noncausal regression models. Based on the SCM, inference of ACE provides substantiated and validated research hypotheses for new products and support for decisions of potential interventions for improvement in product design, new process introduction, process control, management and marketing.

## INTRODUCTION

**Fig. 1:**
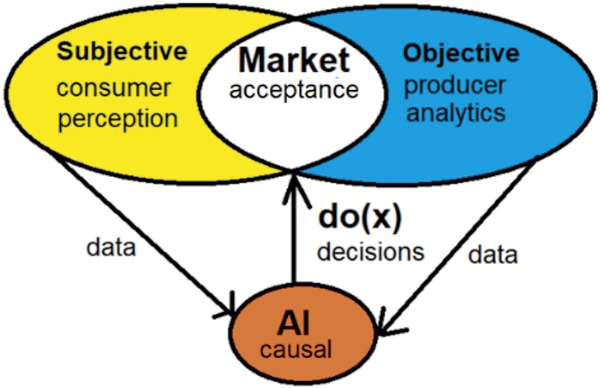
System view of causal AI model application for process and market decision making, management and innovations of food quality by do(x) inference

According to the EU Commission report by Knowledge Centre for Food Fraud and Quality (KC-FFQ) based on 30 000 respondents, 65 % of them perceived food quality as ’very important’ when deciding what to buy, compared to food price, which is important to 54 % of consumers (*1*). The concept of consumer-perceived food quality is very complex, it is an untaggable interaction of the objective measurable physicochemical properties and numerous subjective factors such as consumer population culture, ethical issues, economic and social status, tradition, personal preferences and expected nutritional benefits. It is a multi-dimensional concept which is influenced by a wide range of unmeasurable situational and contextual factors. To food producers, these complexities are difficult to rationalize for possible applications of statistical and mathematical decision-making algorithms. The objective characterization of food complexity can be greatly rationalized using ’big data‘ generated with high throughput analytical instrumentation. Automation of instrument-measurable sensory attributes has led to the development of systems such as electronic noses, e-tongue, near-infrared spectroscopy (NIR), infrared spectroscopy (IR), photoacoustic detectors and computer vision (*2*-*6*). They are applied for online production monitoring, process control and food safety. The fusion of physicochemical and electronic sensory data with computer vision enables the application of machine learning models for the detection of specific signal patterns. It helps food companies in recognizing patterns which drive consumer choice of specific products and improve the chances of continued purchase and potentially in the innovation of new products optimally adjusted to specific markets. Commonly applied statistical models are principal component analysis (PCA) and partial least square regression (PLS), and advanced machine learning (ML) algorithms such as artificial neural networks (ANN), convolution neural networks (CNN), decision trees (DT) and random forests (RF). They analyse large data sets of food quality parameters such as appearance, texture, taste and odour, and identify patterns that may be difficult for humans to detect. Importantly, they can help in identifying food contamination, spoilage and adulteration, which are crucial factors in maintaining food safety. The main benefit of ML models is the ability to provide an ’in-time’ assessment of the statistically significant status of food products (*7*-*10*). Integration of ML models with business knowledge in a food company on a production system level leads to industrial artificial intelligence (AI). It collaborates by supporting and enhancing the human thinking process, enables knowledge management and storing, and most importantly, it can learn. A decade of bibliometric studies on AI related to food science and technology show an exponential increase (*11*). Literature reports indicate that besides academic research, there is also a very strong interest in AI in major companies in the food industry. Dominant industry interest is in the application of intelligent robotics in specific process unit operations and their integration into a whole company AI-supported management. Besides standard engineering applications, AI is becoming a key support in the discovery and introduction of food innovations such as new components for taste, flavour and fragrance, especially aimed to reduce the content of sugar and salt in foods and beverages (*12*). Recent advancements are focused on the integration of strategic decisions of food company policymakers, business intelligence and AI systems in industrial production (*13*-*16*). The success of global integration of AI in food-producing companies depends on understanding the human subjective component of the present and potentially new markets (Fig. 1). Understanding the intricate dependencies of human subjective and objective physicochemical data requires higher levels of AI models as given in the scheme of ’knowledge ladder’ (*17*). Most of the present AI models fit the first knowledge ladder rung with potentially high flexibility and prediction accuracy under unchangeable model training conditions. In a food company, policy decisions of business management and production technologies need AI’s ability to act under new model untrained conditions. Most unforeseen new conditions are due to disruptions of supply chains, effects of climate change on the production of agricultural raw materials, competitor products and shifts in market preferences. The key upgrade of the present AI models is the application of causal data fusion of human subjective and instrumental objective data. Causal AI models are on the second and third rungs of the knowledge ladder. Causal relations are deduced from field knowledge (economy, engineering, physics, chemistry, nutrition) and from big data by statistical and knowledge models. The causal relations are integrated into AI models as Bayesian networks. They enable causal analysis elimination (blocking) of numerous confounding relations present in integrated big data training sets. On the second knowledge rung, causal AI models are applied for predicting average causal effect (ACE) of potential policy decisions and/or production interventions, labelled as do operator do(x) in Fig. 1. On the third rung, causal AI models are applied for counterfactual reasoning approaching human imaginative intelligence (*17*). The aim of this manuscript is to apply causal AI modelling of food quality assessed by consumers and a professional panel of evaluators of wheat baking quality, fermented dairy products and wine quality.

## MATERIALS AND METHODS

### Wheat quality

The baking quality of seven winter wheat cultivars from the Slavonia region in eastern Croatia was analysed. The volume of bread loaf under the standard baking protocol was used as the baking quality test. The cultivars were grown for a period of three years under controlled conditions at the experimental field of the Agricultural Institute Osijek, Croatia. Their quality properties were evaluated by 45 physical, chemical and biochemical variables. Each parameter was determined in triplicate during three consecutive years of cultivation. The measured variables were grouped as 6 indirect quality parameters, 7 farinographic parameters, 5 extensographic parameters and 25 pieces of information from reversed phase-high performance liquid chromatography (RP-HPLC) of gluten proteins. The experiment methodology and the data are available in the published manuscripts (*18*, *19*). All properties are listed as a table of continuous numerical variables. The data were highly correlated and the average absolute Pearson correlation was R=0.41. Principal component analysis of the total data set revealed that the cumulative effect in explaining the total data variance by the first three components was 76.45 % and the first four components accounted for 82.68 %.

### Dairy quality

This dairy dataset contained 1059 samples of consumer quality assessments of fermented dairy products (*20*, *21*). The dataset consisted of 7 variables: pH, temperature, taste, odour, fat, turbidity and colour. Temperature, pH and colour were instrument-measured properties defined as continuous variables. The average and standard deviation for pH was 6.63±1.4, milk pre-treatment temperatures were in the range from 34 to 90 °C, with an average temperature of 44.2 °C. The colour data were determined spectroscopically with low variability of 1.7 % relative standard deviation. The samples of the physical variables have a non-Gaussian probability distribution. Spearman’s rank-order correlation coefficients between temperature and pH, colour and odour were significant with an average value of ρ=0.25, while the ρ correlation between colour and odour is insignificant. The consumer quality evaluation was the ordinal categorical variable with three levels: low, medium and high. Spearman’s rank-order quality grade correlation with temperature, colour and odour was significant, while pH was insignificant.

### Wine quality

The wine quality was a large dataset, 1599 red and 4898 white samples of the Portuguese Vinho Verde wine, characterized by 12 physical and chemical composition data and quality assessments provided by a panel of professional wine tasters (*22*-*24*). The data file is available from the UCI Machine Learning Repository from the University of California at Irvine, USA. The variables were fixed acidity, volatile acidity, citric acid, residual sugar, chlorides, free sulphur dioxide, total sulphur dioxide, density, pH, sulphates and alcohol. The wine compositions were continuous numerical variables and the quality was an ordinal categorical variable with levels 1-10. The variable density was removed from the data set due to its very high variance inflation factor (VIF) since it is a common effect (causal collider) and hence cofounds modelling parameters (*24*). The probability distributions of the variables were approximately Gaussian. The data were highly correlated and the first three principal components for the red and white wines accounted for 99.7 and 99.8 % of the respective variances. Both red and white wines had maximum relative data variability for citric acid of 71 %, given as the ratio of standard deviation and mean value. The maximum Pearson’s correlations of the quality were with the content of alcohol, R=0.48 and 0.44 for the red and white wines respectively. The maximum negative correlation was with volatile acidity, R=-0.39 and -0.19 for the red and white wines respectively.

### Methodology

The basic principles of causal AI modelling are based on the concepts of Bayesian statistics and networks (BN). Bayesian statistics combines prior knowledge (old model) upgraded with new experimental observations (data) in the prediction of a new model. The nature of prior knowledge in modelling includes deductive (known theoretical knowledge) and inductive (empirical structures and model parameters known from previous studies) processes studies. Knowledge of a causal AI model was expressed as a joint probability density function P of the model conditioned on new data. Causal AI modelling is a two-stage process in which the first objective is to determine the structure of a BN graph G, and in the second stage to determine functional causal dependencies between variables followed by estimation of the model parameters θ.

P(model|data)=P(G=graph,θ=parameter|X*=*data) /1/

The two-stage process of structural causal modelling (SCM) was expressed as a product of the corresponding probability density functions:

P(G,θ|X)=P(G|X) P(θ|G,X) /2/

With inferred causal structure G and parameters, θ model posterior distribution was expressed by the basic Bayesian relationship:


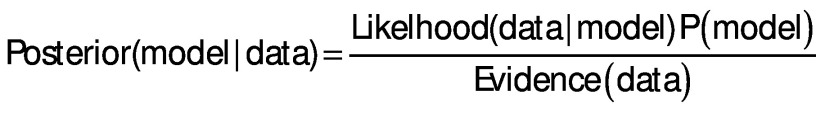
 /3/

In case of a model with continuous random variables (Gaussian), it is explicitly expressed in a functional form as:


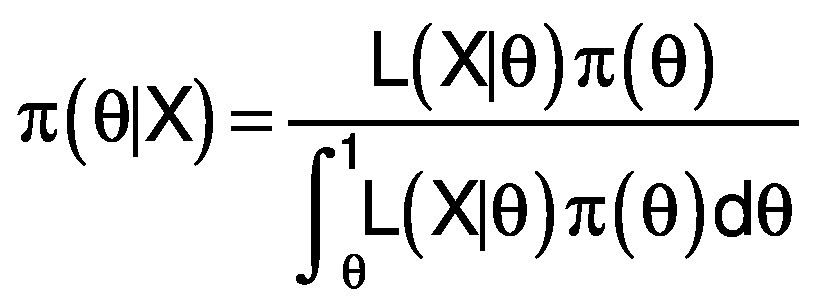
 /4/

Extensive sampling by Monte Carlo Markov chain (MCMC) algorithm was applied for statistical inferences from the model multivariable posterior probability distribution π(θ|X).

Commonly, the basic modelling presumes that all considered causal effects are directional, *i.e.* recurrent causal effects are not considered. It results in model graphs without close loops, which are consequently named directed acyclic graphs (DAG). Markov property of DAG greatly simplifies modelling of complex multivariable stochastic systems (*25*). Complete causal directed acyclic graph G is a set of vertices V (corresponding to the random model variables x_i_) connected with a set E of oriented edges (arrows), G={V,E}. It is a Bayesian network (BN) with Markov property enabling decomposition of a joint probability density function P as a product of individual node (variables x_k_) probabilities p conditioned on their parent variables Pa. The parent variables are those variables x_i_ (vertices) pointing directly to x_k_ *via* a single edge.


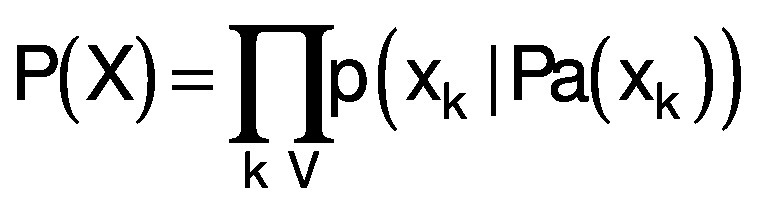
 /5/

Causal dependencies, direct and total, depend on a set of network paths between the cause-and-effect variables. To infer causality, confounding of interfering variables must be blocked by directed d-separation, which implies conditional independence in the probability distribution (*17*). Variables which block interfering interactions define adjustment sets that enable deconfounded (linear and/or nonlinear) estimation of average causal effect (ACE). For models with continuous variables, ACE is evaluated as the derivative of expected value of output variable (effect) Y with respect to the change of input (cause) X at constant covariates, called intervention of cause by do(x) (*6*). In case of a linear SCM, ACE is a value corresponding to average change of effect Y due to the intervention by changing cause X for a unit value. For nonlinear SCM, ACE is a function of the cause X defined by the partial derivative:


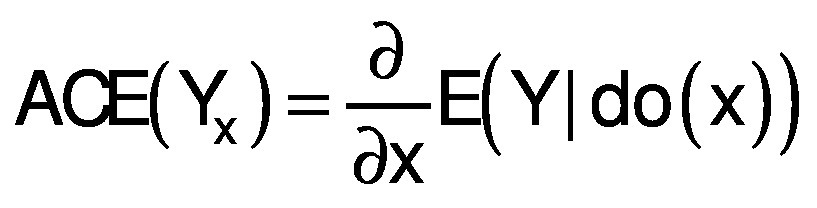
 /6/

## RESULTS AND DISCUSSION

### Wheat baking quality

The wheat data were regularized by the application of a flexible net of least absolute shrinkage and selection operator (LASSO) as a combination of L1 and L2 norm penalty functions (*26*):


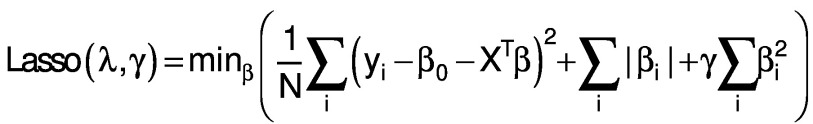
 /7/

The initial space of 45 wheat chemical, physical and biochemical variables was reduced to the space of 10 features obtained by optimisation algorithm provided with glmnet software (*27*-*29*). The selected optimal features were: protein, wet gluten, falling number, water absorption, dough resistance, resistance/extensibility ratio, total glutenin, total high-molecular-mass glutenin, alpha-gliadin and degree of softening of the dough.

**Fig. 2:**
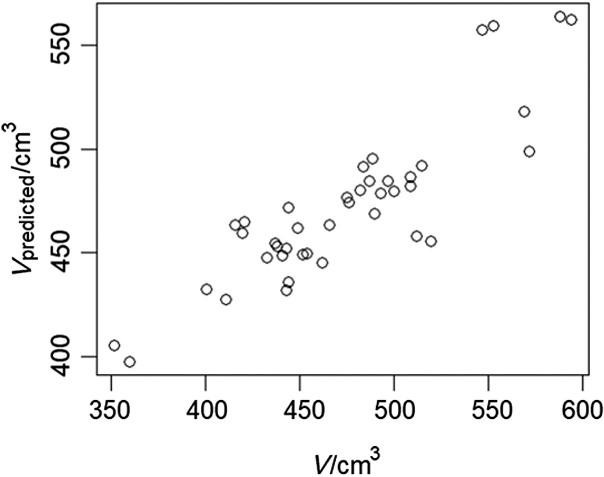
Prediction of the wheat baking quality as volume of product with 10 key features by the random forest model

**Fig. 3:**
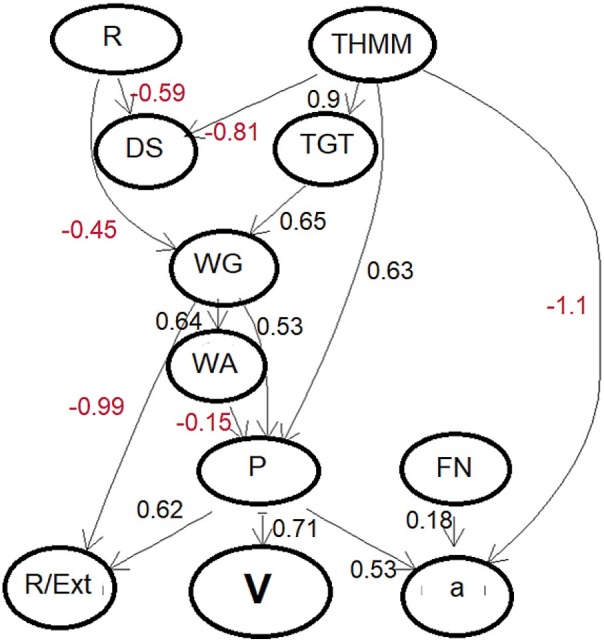
Causal Bayesian network model of the wheat key features and bread baking quality as volume. The path coefficients are the direct causal strengths evaluated with the standardized variables. P=*w*(protein)/%, WG=*w*(wet gluten)/%, FN=falling number/s, V=*V*(bread loaf)/cm^3^, WA=water absorption/%, R=dough resistance/min, R/Ext=resistance/extensibility ratio, TGT=*w*(glutenin)_total_/%, THMM=total high molecular mass/%, a=*w*(α-gliadin)/% and DS=high degree of softening

The model was the assembly of 500 trees, each obtained by random split of 3 variables. Validation of the prediction model showed that with the untrained out-of-bag samples it accounted for 75 % of variance (*30*). Fig. 2 shows the performance of the model predictions. Causal relations between the key variables were evaluated as a directed acyclic graph (DAG). The DAG was shown with the key variables as the nodes, associations between the variables as the edges and the causal dependences as arrows. In the process of causal structural learning, the graph edges and orientations of arrows were considered as random variables with statistical properties estimated by Monte Carlo Markov Chain (MCMC) sampling from Bayesian posterior distribution, provided as BNDAG software support (*31*, *32*). The result was structural causal model (SCM) shown as a graph in Fig. 3. The causal strengths, with positive and negative effects, were given as the path coefficients, which were calculated from corresponding d-separated (directionally separated subgraph) adjustment sets by ordinary least squares (OLS) regression with normalized data (*17*, *33*).

The causal inferences of the SCM were compared (validated) using unstructured causal model with double machine learning (DML) algorithm for estimation of the average causal effect (ACE) (*34*). The effects were estimated as the ratio of covariance and variance of the residuals of volume *V* and k-th variable x_k_ predicted by the corresponding random forest (RF) mode l:


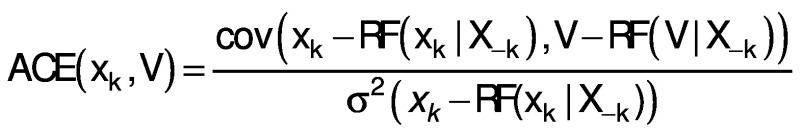
 /8/

**Fig. 4:**
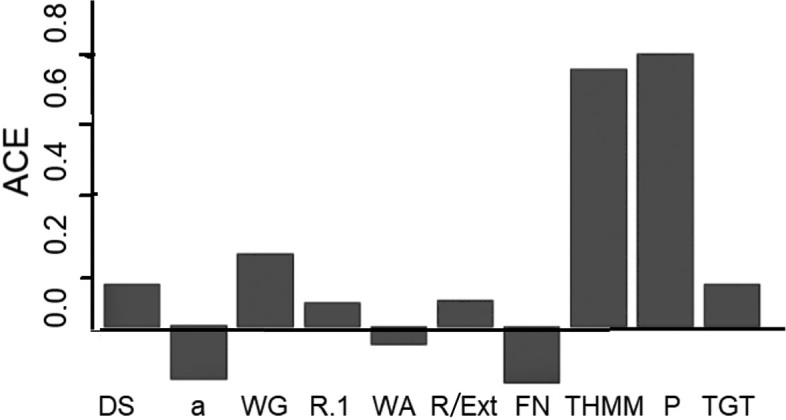
Direct average causal effects (ACE) of the wheat key features on bread baking quality. The ACE values were evaluated with the standardized variables. DS=high degree of softening, a=*w*(α-gliadin)/%, WG=*w*(wet gluten)/%, R.1=dough resistance/min, WA=water absorption/%, R/Ext=resistance/extensibility ratio, FN=falling number/s, THMM=total high molecular mass/%, P=*w*(protein)/% and TGT=*w*(glutenin)_total_/%

The ACE estimates with standardized data are shown as a bar chart (Fig. 4). The SCM and the ACE estimates confirm the dominant positive effects on bread baking quality (V) of protein (P) and total high-molecular-mass (THMM) content.

**Fig. 5:**
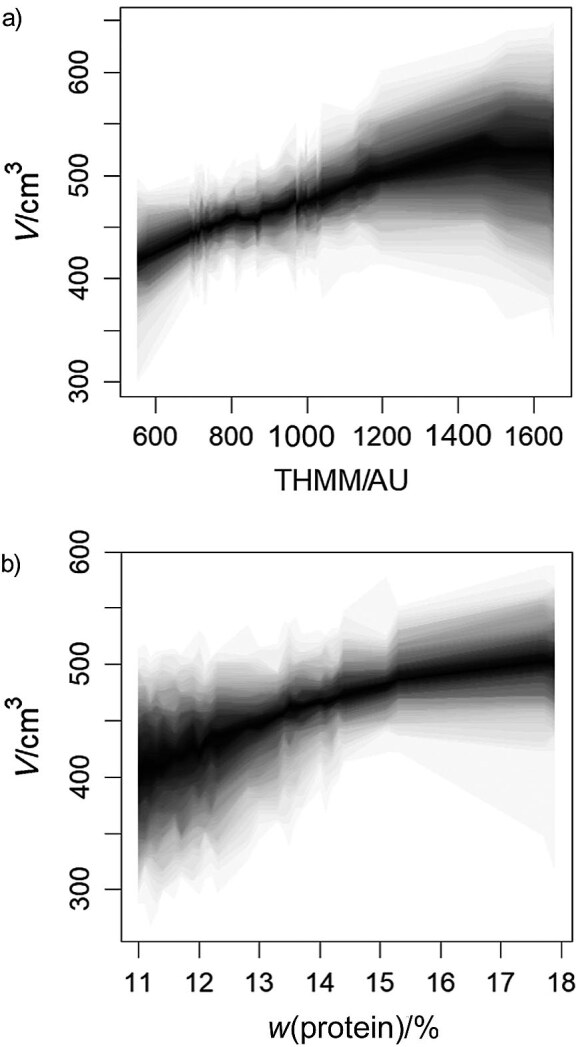
Distributions of a bread loaf volume (do(x)) caused by the intervention of do(x) on the content of: a) total high-molecular-mass (THMM) gliadins and b) protein

The main technological benefit is the application of the SCM to predict unconfounded effects of intervention action, *i.e.* doing effects (*17*). The do(x) operator was applied to redesign original DAG and accordingly modify the joint probability function by replacement of random variable X_k_ with preselected deterministic value x_k_ and d-separation of confounding variables which simultaneously interfere with the intervention (treatment) and effect (outcome). To account for nonlinearity and probability in uncertainty of do(x) effects, Bayesian neural networks (BNN) were developed (*35*). The intervention effects of the key causal variables P and THMM on bread baking quality V are shown in Fig. 5. The distributions of the effect V indicate considerable uncertainty due to the covariates from the adjustment sets and modest saturation type nonlinearities.

### Dairy product quality

**Fig. 6:**
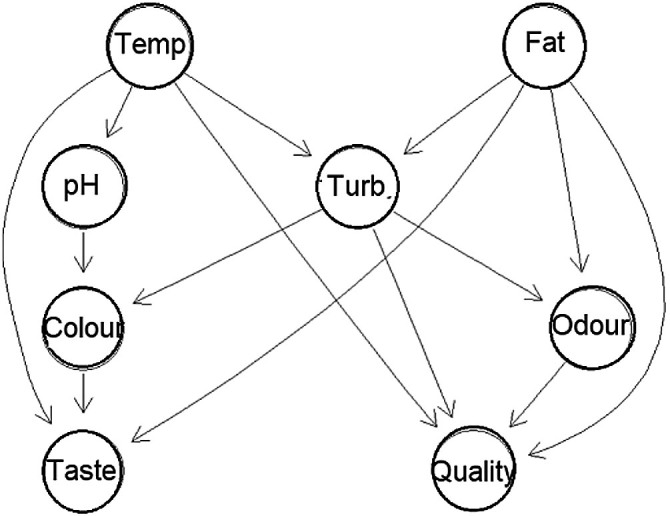
Directed acyclic graph (DAG) of causal effects of milk composition and process parameters on consumer assessment of dairy quality. Temp.=temperature, Turb.=turbidity

**Fig. 7:**
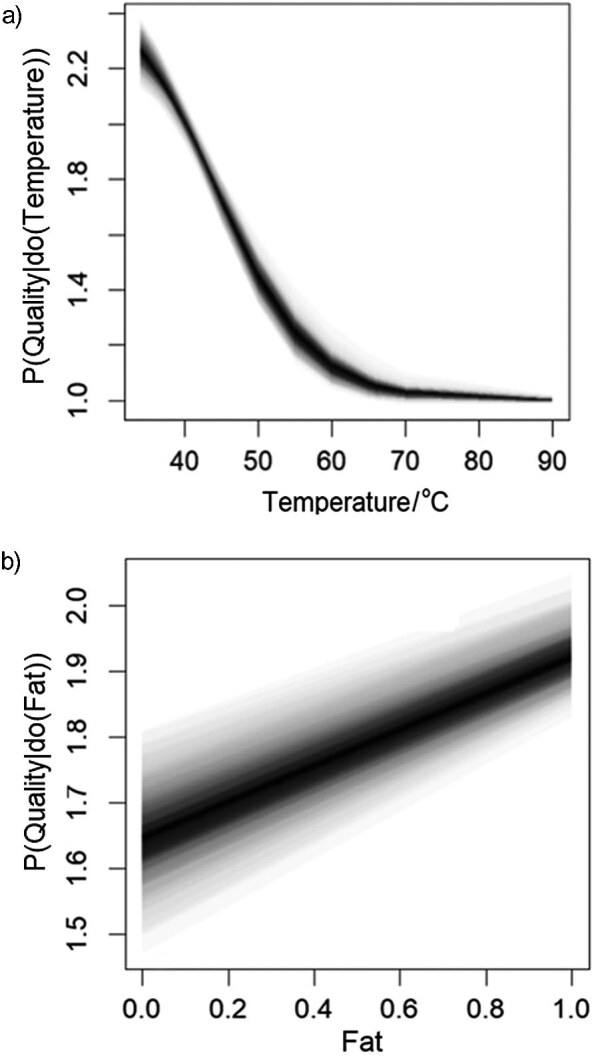
Probability distribution of quality(do(x)) of consumer assessment of dairy quality caused by a change in: a) pretreatment temperature (°C) and b) relative fat content

Causal analysis of the dairy product quality data was based on the SCM. Causal structure network learns by hill-climbing (HC) algorithm of greedy search of DAG space of association structures and causal directions to optimize Bayesian information criterion (BIC) (*36*). A relatively simple DAG network shown in Fig. 6 was obtained. Temperature and fat content were identified as the exogeneous variables, which with product quality and taste are common effects as colliders. The endogenous variables were product pH, odour, turbidity and colour. The product taste and quality grade had common causal ancestors with maximum negative correlation between the grade and temperature of R=-0.45 and maximum positive correlation between the taste and fat content of R=0.32. Predictive power of the random forest model with 500 trees and 2 randomly selected variables at each had very high yield with the average out-of-bag classification error of <1 % (*30*). The maximum causal effect on the quality as negative ACE on temperature was -0.04 quality grade/°C in the temperature range 25–60 °C. The ACE of fat content on quality grade was 0.4. Functional dependences of ACE were obtained using the adjusted d-separated variables of the Bayesian neural network shown as partial dependent plots in Fig. 7. The ACE of temperature was highly nonlinear with the saturation low point at about 60 °C, while the ACE of fat content was positive and linear in the full range.

### Wine quality analysis

**Fig. 8:**
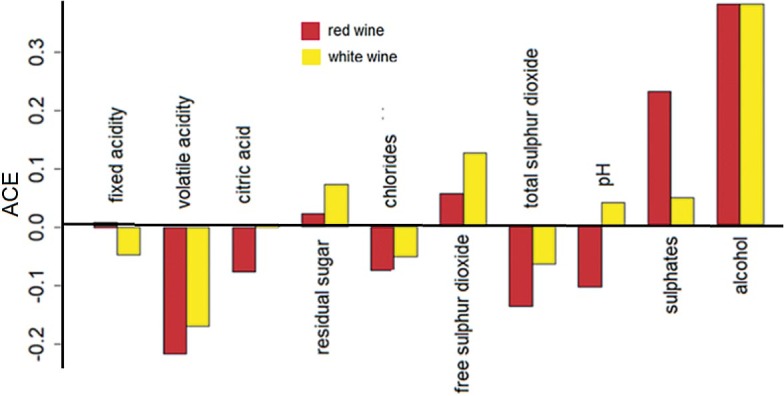
The average causal effect (ACE) of wine quality caused by the change of the standardized values of physical and chemical parameters

For the wine quality detailed description of SCM and causal analysis is given by Kurtanjek (*24*). Here the causal effects were determined by SCM validated by unstructured DML causality model (*34*). The model given in Eq. 8 was applied. The random forest modelling was applied to standardized data sets separately for red and white wines. The models with relative average prediction errors of 5.13 and 4.17 % were obtained for red and white wines respectively. The comparative ACE of the red and white wines are jointly presented in Fig. 8. Alcohol content, predicted by the DML and SCM, had the highest positive ACE on quality of red and white wines. The content of sulphates and free sulphur dioxide had the second most important positive ACE on both red and white wines, while volatile acidity had the highest negative ACE. Although SCM and DML are based on different assumptions, the corresponding ACE estimates were qualitatively and numerically almost in agreement.

## CONCLUSIONS

This manuscript provides methodologies of causal AI modelling applied to complex problem of integration of objective (instrumental) and subjective (human) food quality data. The obtained causal network model helps food engineers with intervention decisions for the existing and innovation of new technologies. The methodologies are illustrated by the models of bread baking quality, fermented dairy products and wine.

Machine learning models of neural networks and random forest of decision trees were applied. The key research objective is discovery of the causal relations between the objective physicochemical data and consumer perception of quality. To find causal relationships between complex data of wheat biochemical and physical properties and bread baking quality, Bayesian statistical model with Monte Carlo Markov chain (MCMC) sampling of the posterior distribution was applied. Structural causal learning and analysis of dairy products was achieved by hill-climbing optimization of the Bayesian information criterion (BIC). Besides the structural causal models, the unstructured algorithm of double machine learning (DML) models with the random forest decision trees were applied to obtain the vine quality data.

The main technological application of the presented causal artificial models is to evaluate the effects of interventions (’do’, do(x) operator) as improvements of production process parameters and compositions of food ingredients. The causal models help find process control patterns and support technological decisions outside the available regression data. Here, for each presented model, average causal effects (ACE) were evaluated based on d-separation criteria and selection of the corresponding unconfounding adjustment sets. For the models to compare wine quality, the structural models based on ACE are in agreement with the estimates by the unstructured DML algorithm. The importance of nonlinear causal effects is modelled by Bayesian neural networks with d-separated minimal adjustment sets and shown as partial dependency plots.
